# A novel 5-enolpyruvoylshikimate-3-phosphate (EPSP) synthase transgene for glyphosate resistance stimulates growth and fecundity in weedy rice (*Oryza sativa*) without herbicide

**DOI:** 10.1111/nph.12428

**Published:** 2013-08-01

**Authors:** Wei Wang, Hui Xia, Xiao Yang, Ting Xu, Hong Jiang Si, Xing Xing Cai, Feng Wang, Jun Su, Allison A Snow, Bao-Rong Lu

**Affiliations:** 1 Ministry of Education Key Laboratory for Biodiversity and Ecological Engineering, Institute of Biodiversity Science, Fudan UniversityHandan Road 220, Shanghai, 200433, China; 2 Fujian Province Key Laboratory of Genetic Engineering for Agriculture, Fujian Academy of Agricultural SciencesFuzhou, 350003, China; 3 Department of Evolution, Ecology, & Organismal Biology, Ohio State UniversityColumbus, OH, 43210-1293, USA

**Keywords:** *epsps*, fitness, genetically engineered, glyphosate-resistant, introgression, *Oryza sativa*, risk assessment, weedy rice

## Abstract

- Understanding evolutionary interactions among crops and weeds can facilitate effective weed management. For example, gene flow from crops to their wild or weedy relatives can lead to rapid evolution in recipient populations. In rice (*Oryza sativa*), transgenic herbicide resistance is expected to spread to conspecific weedy rice (*Oryza sativa* f. *spontanea*) via hybridization.
- Here, we studied fitness effects of transgenic over-expression of a native 5-enolpyruvoylshikimate-3-phosphate synthase (*epsps*) gene developed to confer glyphosate resistance in rice. Controlling for genetic background, we examined physiological traits and field performance of crop–weed hybrid lineages that segregated for the presence or absence of this novel *epsps* transgene.
- Surprisingly, we found that transgenic F_2_ crop–weed hybrids produced 48–125% more seeds per plant than nontransgenic controls in monoculture- and mixed-planting designs without glyphosate application. Transgenic plants also had greater EPSPS protein levels, tryptophan concentrations, photosynthetic rates, and per cent seed germination compared with nontransgenic controls.
- Our findings suggest that over-expression of a native rice *epsps* gene can lead to fitness advantages, even without exposure to glyphosate. We hypothesize that over-expressed *epsps* may be useful to breeders and, if deployed, could result in fitness benefits in weedy relatives following transgene introgression.

## Introduction

Nearly all plants, bacteria, and fungi rely on a 5-enolpyruvoylshikimate-3-phosphate (EPSP) synthase (*epsps*) gene encoding EPSPS (EC2.5.1.19), a key enzyme in the shikimic acid pathway (Herrmann, 1995). This pathway, which is inhibited by the herbicide glyphosate (*N*-(phosphonomethyl)glycine), produces aromatic amino acids, lignin, flavonoids, phenolics, and other secondary metabolites that can account for as much as 35% of a plant's biomass (Shah *et al.*, 1986; Franz *et al.*, 1997). Reduced amounts or functionality of EPSPS disrupt the pathway, leading to effects such as reductions in aromatic amino acids and indole-3-acetic acid (IAA), a master signaling hormone for plant growth and development (Müller *et al.*, 1998), and ultimately causing the plant to weaken and die (Funke *et al.*, 2006).

After glyphosate was widely adopted as a systemic herbicide, researchers discovered resistant microbes with mutant *epsps* genes (*aroA*, where *aro* denotes genes regulating biosynthesis of several aromatic amino acids, and *A* represents a mutation type of the gene (Demerec *et al.*, 2002)), including the CP4 strain of *Agrobacterium* sp. (Sun *et al.*, 2005; Funke *et al.*, 2006; Richards *et al.*, 2006; Jin *et al.*, 2007). Genetically engineered (GE) *aroA* genes from *Agrobacterium* sp. strain CP4 were developed by scientists at Monsanto Company (Missouri, USA) in the 1980s (Funke *et al.*, 2006) and have since been inserted into transgenic, RoundUp Ready® (Monsanto) crops that now occupy millions of hectares world-wide (Green, 2009; Benbrook, 2012; James, 2012). Other methods for identifying genes for glyphosate resistance include site-directed mutagenesis and DNA shuffling/screening, also known as directed evolution (Comai *et al.*, 1983, 1985; Funke *et al.*, 2006; Green, 2009; Tian *et al.*, 2011).

Plant-derived *epsps* genes are known to have the same origin and function as *aroA* genes in microbes (Richards *et al.*, 2006; Xu *et al.*, 2002). For a few years, starting in 1998, Monsanto sold a type of RoundUp Ready® corn (*Zea mays*) known as Event GA21 with a modified *epsps* gene from corn linked to a constitutive actin promoter from rice (*Oryza sativa*) (Monsanto, 2002). The modified EPSPS protein of GA21 had a low affinity for glyphosate and differed from the native EPSPS protein by only two amino acids (Monsanto, 2002), including a mutation at amino acid 106, which has been reported in some glyphosate-resistant weeds (Powles & Preston, 2006).

In contrast to the examples cited above, our current study involved transgenic over-expression of an endogenous *epsps* gene as a mechanism for glyphosate resistance. In previous studies, this mechanism was evaluated as a possible strategy for glyphosate resistance in commercial agriculture (Steinrucken *et al.*, 1986; Klee *et al.*, 1987). Scientists at Monsanto selected glyphosate-resistant cell lines in *Petunia hybrida* that overproduced EPSPS (Steinrucken *et al.*, 1986). They then cloned the *epsps* gene from these lines and inserted it back into *P. hybrida* plants with the cauliflower mosaic virus 35S promoter (Shah *et al.*, 1986). The transgenic cell lines overproduced EPSPS by 15- to 20-fold and gave rise to plants that survived four times the dose of RoundUp® needed to kill nontransformed plants (Shah *et al.*, 1986). Although researchers at Monsanto concluded that over-expression of *epsps* to obtain glyphosate resistance was not sufficiently effective for commercial application (Kishore *et al.*, 1992; Shaner *et al.*, 2011), ongoing research in China suggests that this approach can be very effective in tobacco (*Nicotiana tabacum*) and rice (Xu *et al.*, 2002; Yan *et al.*, 2011; F. Wang, unpublished data).

To our knowledge, possible yield benefits of overproducing EPSPS in plants have not been reported in peer-reviewed journals. Instead, the goal of manipulating *epsps* gene expression has been focused on its utility for developing glyphosate-resistant crops. Thus, remarkably little is known about how the overproduction of EPSPS affects plant growth rates, competitive ability, yield, and lifetime fitness. We became intrigued by this question in the context of our research on ecological and evolutionary effects of transgenes that spread from crops to feral populations or enter wild/weedy populations via hybridization (Lu & Snow, 2005). The identified endogenous *epsps* gene in rice (Xu *et al.*, 2002) allowed us to investigate these questions with crop–wild rice hybrids. Because EPSPS is involved in aromatic amino acid synthesis and other essential functions, we asked whether overproduction of EPSPS would promote the growth of crop–weed rice hybrids in the absence of glyphosate application.

Rice has been extensively studied using transgenic methods to introduce traits such as resistance to insects, herbicides, and viruses (Lu & Snow, 2005; Lu & Yang, 2009; Yang *et al.*, 2011). Many GE rice lines with these and other traits have been developed and tested in field trials. One concern regarding the future commercial cultivation of GE rice is that gene flow to weedy rice (*Oryza sativa* f. *spontanea*) or wild rice (*Oryza rufipogon*) may have unwanted ecological consequences if the transgenes confer a strong fitness advantage and become ubiquitous (Lu & Snow, 2005; Yang *et al.*, 2011). Weedy rice, also known as red rice, is a noxious weed that infests rice fields world-wide. Weedy rice is often managed using flooding and/or herbicides and seldom occurs in other crops or in nonagricultural habitats. Pollen-mediated gene flow from rice to weedy rice has been reported (Chen *et al.*, 2004; Shivrain *et al.*, 2007; Tian *et al.*, 2011; Zuo *et al.*, 2011), and herbicide resistance transgenes in cultivated rice are expected to spread to weedy rice within a few seasons of contact (Gressel, 1999; Zuo *et al.*, 2011).

**Fig 1:**
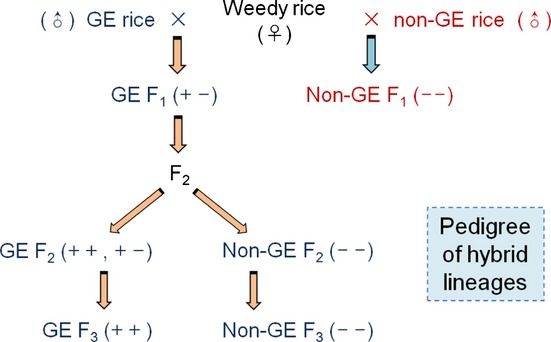
Crossing procedure for obtaining crop–weed (*Oryza sativa* f. *spontanea* and *O. sativa*) hybrid lineages that segregated for the 5-eno-lpyruvoylshikimate-3-phosphate synthase (*epsps*) transgene. Genetically engineered (GE) refers to plants with one (+ −) or two (+ +) copies of the *epsps* transgene. F_1_ and F_2_ plants were used in field experiments to test for differences between GE and non-GE controls in growth and fecundity; F_2_ plants also were used to compare gene expression and EPSPS protein levels; the F_3_ generation was used to test for differences in tryptophan concentration, photosynthetic rate, and per cent seed germination.

Crop genes that confer herbicide resistance are expected to become widespread in weedy rice populations if continued herbicide selection pressure purges nonresistant genotypes from these populations. Therefore, understanding the fitness costs or benefits of a transgene encoding EPSPS and glyphosate resistance in weedy rice is important for assessing potential ecological and agronomic consequences, especially if the transgene also confers a fitness advantage in the absence of glyphosate. We tested for such effects using crop–weed hybrid progeny derived from a GE cultivar and four populations of weedy rice (Fig. 1) in regulated field experiments in China. These crop–weed hybrid lineages segregated for the presence or absence of the novel *epsps* transgene, and transgenic progeny exhibited very strong and consistent increases in plant growth and fecundity compared with their nontransgenic counterparts, without exposure to glyphosate. For brevity, below we refer to the transgene-positive plants as GE and nontransgenic controls as non-GE.

## Materials and Methods

### Plant material

A new herbicide-resistant GE rice (*Oryza sativa* L.) line (EP3), its non-GE rice parent Minghui-86, and four weedy rice (*Oryza sativa* f. *spontanea* Roschevicz) accessions (W1, W2, W3, and W4) were used to generate crop–weed hybrids (Fig. 1; Supporting Information Table S1). The Minghui-86 variety is widely used for rice production and breeding in China. The EP3 transgenic rice, which was derived from Minghui-86, was homozygous for one copy of a modified *epsps* gene cloned from Minghui-63 (Xu *et al.*, 2002) under the control of the maize (*Zea mays*) ubiquitin (*Ubi*) promoter, and tightly linked with a selectable marker gene (hygromycin phosphotransferase, *htp*) for hygromycin resistance. [Correction added after online publication 5 September 2013 to remedy a typographical error in the text: ‘Minghui-63’ has been corrected to ‘Minghui-86’.] This line was generated via *Agrobacterium-*mediated transformation and selfed through the T5 generation. These plants are resistant to glyphosate (F. Wang, unpublished data) and may be developed for commercial rice production in the future. In the crosses described in the following section, the transgene showed expected Mendelian segregation ratios for a dominant trait (B.-R. Lu, unpublished data).

The four weedy rice accessions, which we refer to as biotypes, were collected from Vietnam (W1), South Korea (W2), Nepal (W3), and China (W4) (Table S1). Although only one biotype (W4) was field-tested in its country of origin, the strong and generally consistent effects of the *epsps* transgene across biotypes suggest that similar results would be obtained in other regions.

### Crop–weed hybrid lineages

Crop–weed hybrids represent an excellent system for testing for fitness consequences of transgenes because the F_2_ and subsequent progeny segregate for the presence or absence of the transgene of interest. Thus, GE and non-GE progeny have similar genetic backgrounds, differing only in the presence of the transgene and any traits linked to the transgene insertion site. Here, crop–weed F_1_ hybrids were produced by hand-pollination of weedy rice biotypes (*c*. 20 plants per biotype) with pollen from GE rice (homozygous for the *epsps* transgene) or non-GE rice (with only the natural *epsps* gene), respectively (Fig. 1). Hybrid lineages from the four biotypes were designated as WH1, WH2, WH3, and WH4. F_2_ seeds were obtained by bagging > 100 field-grown F_1_ hybrids that were hemizygous for the transgene to obtain selfed seeds for the GE and non-GE lineages (Fig. 1).

Molecular markers were used to confirm the crop–weed hybrid status of F_1_ plants and determine the GE status of plants in the F_2_ and F_3_ generations. For F_1_ crosses between the GE rice line and weedy rice, PCR of the target transgenic *epsps* with a size of 700 bp and the normal *epsps* gene with a size of 1000 bp (with introns) was conducted using the primer pair 5′-gcagttggaccatcagcgaag-3′, 5′-ctgttgagaaggatgcgaaaga-3′. [Correction added after online publication 5 September 2013. In the original text the authors swapped the primers for RT-PCR and real time PCR. This has been corrected.] Plants with both fragment sizes represented crop–weed hybrids. To identify hybrids from crosses between the non-GE rice line and weedy rice, we used simple sequence repeat (SSR) markers that differed at three loci (RM249, RM307 and RM316, here RM denotes rice microsatellite). PCR analyses of the target transgenic *epsps* gene were used to determine GE and non-GE F_2_ plants, as described above for identifying F_1_ hybrids containing the target *epsps* transgene. The GE F_2_ progeny included both hemizygous and homozygous plants for the *epsps* transgene, whereas all F_3_ progeny were obtained from selfing F_2_ plants that were homozygous for the transgene (based on progeny screening).

### Expression of the *epsps* gene

F_2_ GE versus non-GE plants were compared to test for expected differences in expression of the *epsps* gene using three complementary methods: reverse transcription–PCR (RT-PCR), real-time PCR, and enzyme-linked immunosorbent assays (ELISAs), which measure EPSPS protein levels. For the first two analyses, we used 4-wk-old, glasshouse-grown F_2_ plants from lineage WH1. Three different GE and non-GE plants were used for each of these two analyses. For the ELISAs, we used an independent set of nine F_2_ plants from each GE versus non-GE lineage of WH1, WH2, WH3, and WH4. These glasshouse-grown plants were measured at *c*. 60 d after seeds were sown.

For RT-PCR, total RNA was isolated from leaf tissues using an RNAprep pure plant kit and treated with RNase-free DNase I (TianGen, Beijing, China). After phenol-chloroform extraction and ethanol precipitation, the RNAs were dissolved in diethylpyocarbonate (DEPC)-treated water and reverse-transcribed with the QuantScript RT Kit according to the manufacturer's protocols (TianGen).

All primers used in this study were designed using primer premier 5 (Premier Biosoft International, Palo Alto, CA, USA) and synthesized by Sangon (Shanghai, China). Primer sequences for RT-PCR were: 5′-gcagttggaccatcagcgaag-3′, 5′-ctgttgagaaggatgcgaaaga-3′; for real time PCR: 5′-gagaaggatgcgaaagagg-3′, 5′-acccgacaaccaagtcacca-3′; and 5′-tccatcttggcatctctcag-3′, 5′-gtacccgcat-caggcatctg-3′ for actin. [Correction added after online publication 5 September 2013. In the original text the authors swapped the primers for RT-PCR and real time PCR. This has been corrected in the preceding line.] For each PCR, 1 μl of template cDNA, equivalent to *c*. 100 pg of total RNA, was mixed with 12.5 μl of SYBR Green PCR Master Mix (Applied Biosystems, Warringtion, UK) and 5 pmol each of the forward and reverse primers in a final volume of 25 μl. For RT-PCR, a modified protocol of the ABI 2720 (ABI Company, CA, USA) was used: 30 s at 94°C, 30 s at 58°C and 40 s at 72°C for 30 cycles. RT-PCR product sizes were visualized with electrophoresis using 1.2% agarose. For real-time PCR, the standard protocol of the ABI 7300 Sequence Amplification System was used: 15 s at 95°C and 1 min at 60°C for 40 cycles, followed by the thermal denaturing step to generate the dissociation curves to verify amplification specificity.

For the ELISA analyses, we used the Quantiplate kit (Envirologix, Portland, OR, USA) for detection of the EPSPS protein, according to the manufacturer's protocols. We set the wavelength of the microtiter plate reader to 450 nanometers (nm) using Plate Reader (Bio-Rad Laboratories, Inc., Hercules, CA, USA). We used microplate manager (mpm) software ver. 6 (Bio-Rad Laboratories, Inc.) to summarize the results.

### Measurement of tryptophan concentration

Tryptophan (Trp) was chosen as a representative aromatic amino acid for further study to test for expected differences between GE and non-GE controls. Trp concentration was measured in the F_3_ generation. Nine randomly chosen, glasshouse-grown seedlings at about the four-leaf stage from each GE and non-GE hybrid lineage were measured at the Instrumental Analysis Center of Shanghai Jiao Tong University, Shanghai, China.

The concentration of free Trp (not including proteins) was measured in leaf powder obtained from a liquid nitrogen treatment. Each measurement was made on a mixture of equal quantities of leaf samples from three plants; therefore, three measurements were made for each hybrid combination. One gram of leaf powder was added to a flask containing 50 ml of sodium hydroxide solution (NaOH) (0.02 M), which was heated to boiling and maintained for 1 h. After cooling to room temperature, the solution was slowly filtered and double-distilled water was added to a volume of 100 ml. Twenty milliliters of this solution was placed in a bottle for vacuum drying by a vacuum rotary drier. The dry matter thus obtained was added to 1 ml of hydrochloric acid (HCl) (2 M), and then 5% sulfosalicylic acid was added to completely dissolve the dry matter, which took 20 min at −20°C; the solution was then centrifuged (13 684 ***g***) for 30 min at 4°C. One milliliter of extracted supernatant was filtered through a 0.45-μm polyethersulfone membrane, and adjusted to pH 2. Trp concentration was measured using the automatic amino acid analyzer L-8900 (Hitachi Construction Machinery Co., Ltd, Tokyo, Japan).

### Measurement of photosynthetic rates

Leaf photosynthetic rates (net CO_2_ assimilation in μmol m^−2^ s^−1^) were measured with a portable photosynthesis system (LI-6400; Li-Cor Biosciences, Lincoln, NE, USA) in January 2011. We used glasshouse-grown GE and non-GE F_3_ plants (*n* = 3) from each of the four crop–weed hybrid lineages. This factorial design allowed us to determine the effects of the transgene (GE versus non-GE), the weedy hybrid lineage (WH1–WH4) and their interaction on photosynthesis. Although it would have been useful to measure photosynthetic rates under field conditions as well, this glasshouse experiment allowed us to compare the relative performances of GE and non-GE plants at an early growth stage under uniform conditions.

For each of the eight treatment combinations, three randomly selected seedlings at about the four-leaf stage were measured at Fudan University, Shanghai, using standard protocols for the LI-6400. For each plant, one leaf was measured at four light intensities: 500, 800, 1000, and 1200 μmol m^−2^ s^−1^ of photosynthetically active radiation (PAR). Ambient CO_2_ entering the cuvette was *c*. 400 μl^−1^. In most cases, light saturation occurred near PAR of 1000 μmol m^−2^ s^−1^, but varied somewhat among plants, with some individuals exhibiting signs of photoinhibition at PAR above 800 μmol m^−2^ s^−1^. For statistical comparisons, we recorded the peak photosynthetic rate of each plant across the range of PAR. Thus, statistical analyses (see the Statistical analyses section) are based on one data point from each of three plants in each of the eight treatment combinations.

### Seed germination experiment

GE and non-GE F_3_ seeds derived from the F_2_ hybrid lineages were examined to test for differences in per cent germination. Seeds were germinated on wet filter paper in Petri dishes. For each treatment combination, five replicate Petri dishes, each containing 200 seeds, were placed in a growth chamber at *c*. 37°C and scored when seed germination was complete. Like the crop, the parental weedy rice biotypes do not exhibit seed dormancy (data not shown).

### Field experiments in 2009 and 2011

Common garden experiments were conducted at designated Biosafety Assessment Centers in Fuzhou, Fujian Province, China, in 2009 for F_1_ progeny and in 2011 for F_2_ progeny. The design of these experiments was similar to those in our previous studies (Yang *et al.*, 2011). Each treatment included four replicate plots in 2009 and six replicate plots in 2011. Two cultivation modes were used: pure planting of each set of plants in a plot (36 plants per plot), or mixed planting of GE and non-GE hybrids from the same weedy rice lineage, planted alternately (also a total of 36 plants per plot). The mixed planting treatment was included to increase the likelihood of detecting small fitness differences that might be evident only with direct competition between GE plants and their non-GE counterparts. In both years, 36 plants were planted in a 6 × 6 grid with 20 cm between plants in each replicate plot. The field layout of all treatments was arranged in a randomized design.

Each year, young plants (*c*. 4 wk old) were transplanted from the outdoor nursery to the common garden in early April and harvested at maturity in July. During the 4 wk in the nursery, the plants were randomly positioned. Cultivation management during the field experiment followed procedures used routinely by local farmers. Approximately 10 d before transplanting, the field plots were treated with paraquat (N,N′-dimethyl-4,4′-bipyridinium dichloride) to remove weeds and the field plots were flooded 2 d before transplanting to remove potential paraquat residues. After transplanting, weeds were removed by hand-weeding. Approximately 1–1.2 kg of urea (nitrogen) per 100 m^2^ was applied 1 wk after transplanting. Insecticides that are commonly used in rice fields were applied as needed to control insect pests (i.e. O,S-dimethylphosphoramidothioate; sodium S-(2-(dimethylamino)-3-(sulfosulfanyl)propyl) sulfurothioat; O,O-dimethyl methylcarbamoylmethyl phosphorodithioate; and 2-(tert-butylimino)-3-isopropyl-5-phenylperhydro-1,3,5-thidiazin-4-1). To avoid seed loss caused by seed shattering, all panicles of each plant were enclosed in a nylon bag 10 d after flowering.

Six fitness-related traits were measured, including the total number of seeds per plant, number of tillers, number of panicles, height, per cent seed set, and 1000-seed weight (Table S2). Thirty-six plants in each pure planting plot and 18 plants of each type in each mixed planting plot were measured and these data were averaged to obtain one measurement per plot for each trait.

### Statistical analyses

For the field experiments, data from the pure and mixed planting treatments were analyzed separately. ANOVAs were carried out for the pure treatment because each observation was independent of the others, while paired *t*-tests with Bonferroni corrections were used for the mixed planting treatments to test for significant differences between GE and corresponding non-GE plants. In the ANOVAs, we tested for significant effects of the transgene (as a fixed effect) and the four weedy hybrid lineages (as a random effect). For all comparisons involving pairs of means (GE versus non-GE), we used independent *t*-tests for pure cultivation and paired *t*-tests for mixed cultivation with Bonferroni corrections. Similar analyses were carried out for data on seed germination, Trp concentration, photosynthetic rate, and EPSPS content. Statistical analyses were performed using the software package spss ver. 19.0 for Windows (IBM Inc., New York, USA).

## Results

The *epsps* transgene from rice with the ubiquitin promoter from maize was associated with major increases in transgene expression, Trp concentration, photosynthetic rate, per cent seed germination, plant growth, and fecundity in crop–weed rice hybrids. The GE plants consistently performed better than their non-GE counterparts without herbicide application (Tables2, S3; Figs5).

**Table 1:** Two-way ANOVAs to test the effects of the 5-enolpyruvoylshikimate-3-phosphate synthase (*epsps*) transgene (fixed effect), four weedy rice (*Oryza sativa* f. *spontanea*) biotypes (random effect), and their interaction on EPSPS content (F_2_ generation), tryptophan concentration (F_3_), photosynthetic rate (F_3_), and seed germination (F_3_)

Trait	Transgene (T)			Weedy rice (W)			T × W		
	df	*F*	*P*	df	*F*	*P*	df	*F*	*P*
EPSPS content	1	8.289	0.064	3	1.635	0.345	3	3.351	0.045
Tryptophan concentration	1	14.996	0.030	3	8.604	0.055	3	5.092	0.012
Photosynthetic rate	1	118.369	0.002	3	14.536	0.027	3	0.395	0.758
Seed germination (%)	1	25.773	0.015	3	5.657	0.094	3	3.317	0.032

The transgene (T) had two levels (genetically engineered (GE) or non-GE) and weedy rice biotype (W) had four levels.

**Table 2:** Two-way ANOVAs to test the effects of the 5-enolpyruvoylshikimate-3-phosphate synthase (*epsps*) transgene (fixed effect), four weedy rice (*Oryza sativa* f. *spontanea*) biotypes (random effect), and their interaction on fitness-related traits in F_1_ (a) and F_2_ (b) crop–weed (*Oryza sativa* f. *spontanea* and *Oryza sativa*) hybrids

Trait	Transgene (T)			Weedy rice (W)			T × W		
	df	*F*	*P*	df	*F*	*P*	df	*F*	*P*
(a) F_1_ generation of crop–weed hybrids									
Plant height (cm)	1	0.06	0.816	3	0.29	0.828	3	2.38	0.077
No. of tillers per plant	1	27.84	0.011	3	24.85	0.016	3	4.85	0.009
No. of panicles per plant	1	19.03	0.022	3	23.17	0.014	3	5.53	0.005
No. of seeds per plant	1	47.61	0.006	3	2.60	0.226	3	4.58	0.011
Seed set (%)	1	9.04	0.057	3	0.26	0.852	3	1.99	0.143
1000-seed weight (g)	1	1.86	0.266	3	11.02	0.040	3	0.80	0.505
(b) F_2_ generation of crop–weed hybrids									
Plant height (cm)	1	0.037	0.860	3	26.05	0.012	3	0.34	0.794
No. of tillers per plant	1	29.16	0.012	3	26.95	0.011	3	4.28	0.010
No. of panicles per plant	1	19.84	0.021	3	24.12	0.013	3	6.79	0.001
No. of seeds per plant	1	381.36	0.000	3	9.29	0.050	3	0.46	0.709
Seed set (%)	1	4.99	0.112	3	3.45	0.168	3	1.89	0.147
1000-seed weight (g)	1	8.782	0.059	3	181.82	0.001	3	0.09	0.967

The transgene (T) had two levels (genetically engineered (GE) or non-GE) and weedy rice biotype (W) had four levels.

**Fig 2:**
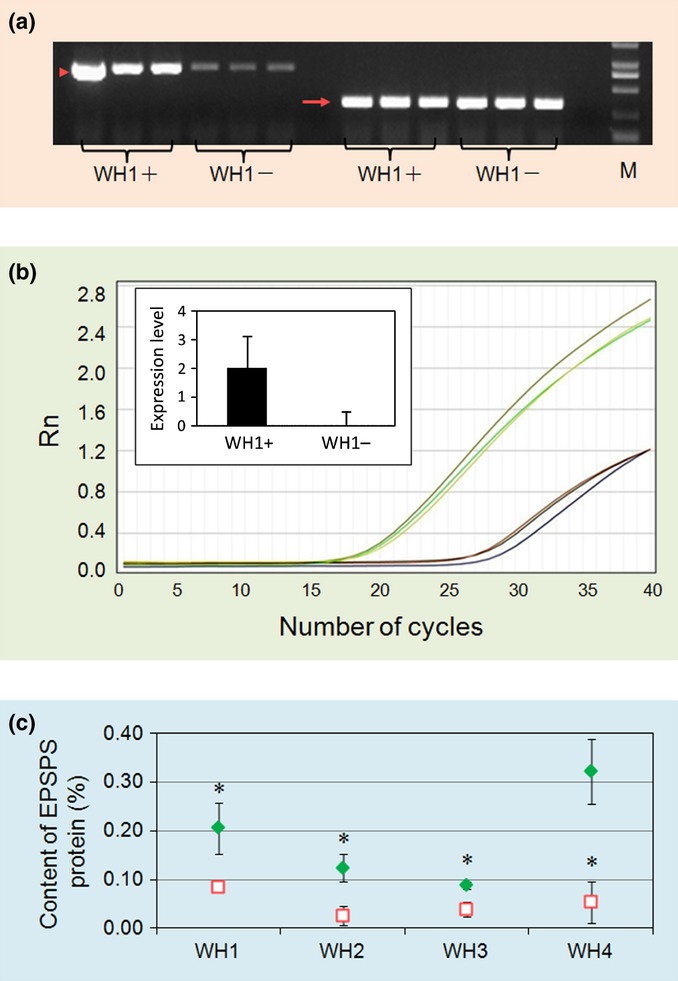
Increased expression of the 5-enolpyruvoylshikimate-3-phosphate synthase (*epsps*) gene and EPSPS protein content in transgenic F_2_ crop–weed (*Oryza sativa* f. *spontanea* and *O. sativa*) hybrids. The four crop–weed hybrid lineages are designated WH1, WH2, WH3, and WH4. Means are shown with 1 ± SE. *, *P* < 0.05; **, *P* < 0.01; ***, *P* < 0.001 (*t*-tests with Bonferroni corrections). (a) Gene expression from *epsps* (arrowhead, left side) and actin control (full arrow, right side) in genetically engineered (GE) (WH1+) and non-GE (WH1−) plants; M indicates the DL2000 DNA marker; *n* = 3. (b) *epsps* gene expression in GE (upper lines) and non-GE (lower lines) WH1 plants detected by real-time PCR. Rn represents the amount of PCR produced by the epsps transgene against that by a reference gene (action) as measured based on fluorescence intensity, shown with the number of PCR cycles. The inserted bar chart represents the relative levels of gene expression when WH1− was set as 1. The *y*-axis shows the logarithm-transformed (log_10_) gene expression level; *n* = 3. (c) Mean percentage of relative EPSPS protein content of GE (green symbols) and non-GE plants (red symbols), determined by enzyme-linked immunosorbent assay (ELISA); *n* = 9.

**Fig 3:**
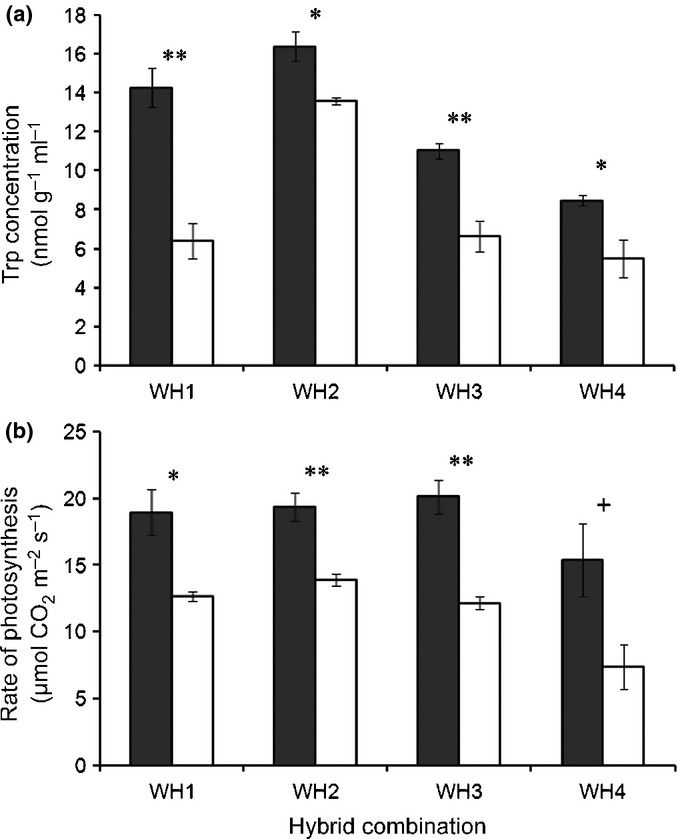
Increases in (a) tryptophan (Trp) concentration and (b) photosynthetic rates of transgenic F_3_ plants from four crop–weed (*Oryza sativa* f. *spontanea* and *Oryza sativa*) hybrid lineages. Closed columns indicate genetically engineered (GE) plants and open columns indicate non-GE plants. The four crop–weed hybrid lineages are designated WH1, WH2, WH3, and WH4. Means and SE are based on *n* = 3 leaf samples for Trp and *n* = 3 plants for photosynthesis. +, *P* < 0.1; *, *P* < 0.05; **, *P* < 0.01 (*t*-tests with Bonferroni corrections). Error bars represent ± SE.

**Fig 4:**
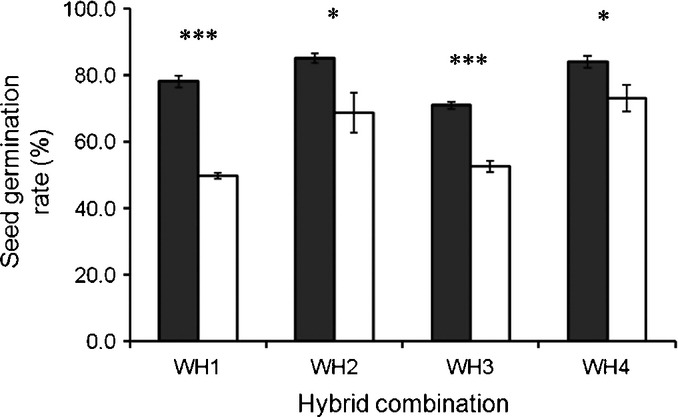
Increased per cent germination of F_3_ seeds with the 5-enolpyruvoylshikimate-3-phosphate synthase (*epsps*) transgene in each crop–weed (*Oryza sativa* f. *spontanea* and *Oryza sativa*) hybrid lineage. The four crop–weed hybrid lineages are designated WH1, WH2, WH3, and WH4. Closed columns indicate genetically engineered (GE) and open columns indicate non-GE plants. *n* = 4 and the error bars represent ± SE. *, *P* < 0.05; ***, *P* < 0.001 (*t*-tests with Bonferroni corrections).

**Fig 5:**
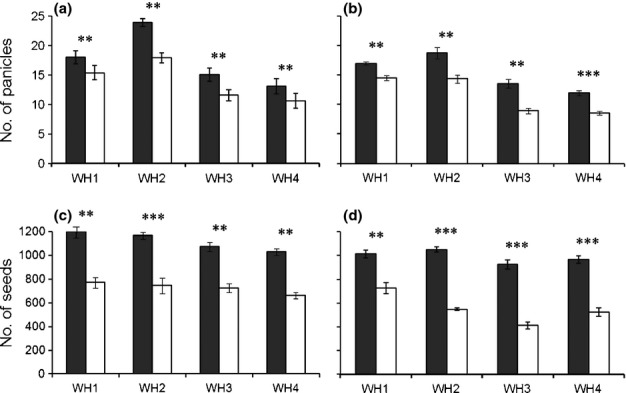
Increased growth and fecundity of F_2_ crop–weed (*Oryza sativa* f. *spontanea* and *Oryza sativa*) hybrid progeny with the 5-enolpyruvoylshikimate-3-phosphate synthase (*epsps*) transgene in field experiments. The number of panicles (flowering shoots) and seeds per plant for each of the four crop–weed lineages are shown, with means and SE (*n* = 6). Closed columns indicate genetically engineered (GE) plants and open columns indicate non-GE plants. (a, c) Results from pure cultivation of either GE or non-GE plants in each plot (compared using *t*-tests with Bonferroni corrections); (b, d) results from mixed cultivation of GE and non-GE plants competing in the same plot (compared using paired *t*-tests with Bonferroni corrections). **, *P* < 0.01; ***, *P* < 0.001; see Table 2 for ANOVAs and Supporting Information Table S3 for a summary of these and other fitness-related traits.

### Expression of the *epsps* gene

As expected, GE F_2_ progeny had much higher expression of the *epsps* gene than their non-GE counterparts (Fig. 2a,b; similar differences in expression were obtained for seedlings and bolting plants (data not shown)). Also, GE plants significantly overproduced the EPSPS enzyme in all four F_2_ crop–weed hybrid lineages (Table 1; Fig. 2c). Results from these independent comparisons, each based on a separate group of plants, confirm that the expression of the *epsps* transgene was much higher than that of the endogenous *epsps* gene of non-GE control plants.

### Tryptophan concentrations and photosynthesis rates

The transgene was also associated with significantly greater concentrations of Trp, which is a downstream product of EPSPS, in comparisons with non-GE F_3_ plants (Table 1; Fig. 3a). The magnitude of this increase ranged from an average of > 100% in WH1 to *c*. 20% in WH2, and a two-way ANOVA showed significant effects of the transgene, weedy rice biotype, and their interaction on Trp concentrations (Table 1). In addition, using 30-d-old, glasshouse-grown plants, we compared photosynthetic rates of segregating GE and non-GE plants from the F_3_ generation and found that photosynthetic rates were *c*. 40–109% greater for GE plants in each of the four hybrid lineages compared with their non-GE counterparts (Fig. 3b). A two-way ANOVA showed significant effects of the transgene and weedy rice biotype on photosynthetic rate (Table 1).

### Fitness-related traits

These physiological effects of the *epsps* transgene were associated with dramatic changes in fitness-related traits. When F_3_ seeds were incubated under favorable conditions for germination, the final per cent germination was as much as *c*. 15–55% greater for GE progeny in each of the four crop–weed lineages, especially in WH1 and WH3, compared with the non-GE progeny (Table 1; Fig. 4).

In field experiments carried out in 2009 and 2011, we observed significant increases in the numbers of tillers and flowering panicles per plant, compared with non-GE controls in each crop–weed hybrid lineage (Tables2, S3; Fig. 5). Beneficial effects of the *epsps* transgene on plant growth resulted in dramatic differences in seed production (Tables2, S3; Fig. 5), which is a key fitness component for annual weedy rice. In 2011, GE F_2_ crop–weed hybrids from the four lineages of weedy rice produced 48–57% more seeds per plant than non-GE controls in pure monoculture plots, and 85–125% more seeds per plant in mixed plots, where direct competition favored the GE plants at the expense of the non-GE plants (Table S3; Fig. 5). Similar results were obtained in the F_1_ generation in 2009 (Table S3). For some traits, the interaction between transgene and weedy biotype was significant (Table 2), reflecting a stronger growth response in some weedy biotypes than others (Table S3). Effects of the transgene on plant height, per cent seed set, and 1000-seed weight were generally nonsignificant or marginally significant (Tables2, S3). In no case did we detect evidence for fitness costs associated with the transgene.

## Discussion

### Fitness benefit of the transgene in crop–weed hybrids without glyphosate application

Originally, this *epsps* transgene was developed to confer resistance to glyphosate, but it also appears to provide profound and previously unrecognized pleiotropic benefits for plant growth and seed production. Given the consistency of our results, we assume that the overproduction of EPSPS and the downstream differences that we observed between GE plants and their non-GE counterparts were attributable to the over-expression of the modified transgene, *epsps*, rather than other tightly linked genes from the cultivated parent, although this possibility cannot be ruled out entirely. In the F_2_ and F_3_ generations, the only difference between GE and non-GE crop–weed progeny was the presence or absence of the inserted *epsps* construct (Fig. 1), as well as the selectable marker gene and any crop-specific genes that may be linked to the transgene insertion site. The F_1_ generation included progeny from two very similar crop donors, one GE and the other not, and very similar benefits were associated with the novel transgene compared with the F_2_ and F_3_ generations.

Because the shikimic acid pathway affects carbon flow and biosynthesis, the over-expression of *epsps* may have many interrelated effects on plant growth and development. It is noteworthy that the GE plants in our study also had increased concentrations of the key amino acid Trp, which is a precursor of the growth hormone IAA and many other compounds (Müller *et al.*, 1998). Other products and secondary metabolites were probably also affected, but were not measured here. We also found that GE F_3_ plants had greater photosynthetic rates in the glasshouse, and increased levels of seed germination in a growth chamber experiment compared with non-GE controls.

Results from field experiments further demonstrate beneficial effects of the *epsps* transgene. During two growing seasons, we found enhanced production of tillers, panicles, and seeds in transgenic segregants, probably as a result of the cascading effects of overproduction of EPSPS. Remarkably, the *epsps* transgene was associated with increases of 48–57% in total seeds per plant of F_2_ crop–weed lineages when grown in pure cultivation (Table S3; Fig. 5c); even greater benefits of the transgene were seen in the mixed cultivation plots (Table S3; Fig. 5d). Taken together, our findings indicate that if the modified *epsps* gene is commercialized in cultivated rice without specific mitigation procedures (Gressel, 1999; Gressel & Valverde, 2009; Liu *et al.*, 2012), it may spread to weedy rice populations, persist, and increase in frequency, even in the absence of exposure to glyphosate. Further life-cycle ecological research is needed to examine whether weedy rice biotypes that overproduce EPSPS could have accelerated population growth rates and enhanced competitive ability relative to existing biotypes in the absence of glyphosate exposure, as we surmise.

We do not know whether weedy rice is unique in its response to the *epsps* transgene or whether the fitness benefits we observed depend on relatively luxurious growing conditions with abundant nutrients and water to sustain rapid growth. Moreover, we do not know how other stages of the life cycle, such as seed longevity or seedling establishment, are affected by the *epsps* transgene. Nonetheless, the very strong and positive effects we documented on plant growth and seed production suggest that fitness benefits associated with the *epsps* transgene may be substantial. Further research focusing on the physiological mechanisms by which this occurs would be useful.

### Broader implications

In the near future, we expect that transgenic crop species that over-express the *epsps* gene will be developed commercially to obtain glyphosate resistance and/or improved agronomic performance. In the USA, The Scotts Company (Marysville, OH, USA) is developing a glyphosate-resistant Kentucky bluegrass, *Poa pratensis*, with an *epsps* gene from *Arabidopsis thaliana* driven by a ubiquitin promoter from rice (*Oryza sativa*). This cultivar is not considered to be a plant pest by the US Department of Agriculture (USDA) and is exempt from regulatory oversight in the USA (Blume *et al.* 2010; USDA, 2011a,b). To our knowledge, no details about *epsps* expression in this cultivar have been made public. We recommend that future biotech risk assessments should consider whether new cultivars with modified *epsps* transgenes overproduce EPSPS and therefore might have enhanced growth and greater fitness in feral or crop–wild progeny. It is possible that current glyphosate-resistant crops have enhanced levels of EPSPS relative to control lines, as a result of having strong promoters, but little or no data are available on this topic. For example, glyphosate-tolerant GA21 maize was reported to produce significantly more EPSPS protein than the non-GE control (EFSA, 2007).

Ongoing crop-to-weed gene flow from existing glyphosate-resistant crops mainly involves the CP4 *aroA* gene from *Agrobacterium* or other modified *aroA* genes (Green, 2009), rather than an over-expressed native *epsps* gene from a crop. In some crops, two tandem *epsps* genes have been inserted together, each with a different promoter (Green, 2009). To date, transgenic resistance from CP4 has spread to feral or hybrid populations of creeping bentgrass (*Agrostis* spp.) (Watrud *et al.*, 2004; Zapiola *et al.*, 2008), *Brassica rapa* (Warwick *et al.*, 2008), canola (*Brassica napus*) (Schafer *et al.*, 2011), and alfalfa (*Medicago sativa*) (Greene, 2012). Further research could focus on whether currently used *epsps* transgenes are associated with greater levels of EPSPS and, if so, whether this trait is associated with enhanced fitness in these and other feral or crop–weed hybrid populations, in the absence of exposure to glyphosate.

Although pollen- and seed-mediated gene flow can have rapid and direct evolutionary effects on weedy relatives of crop plants, a more widespread agronomic problem is the fact that at least 24 weed species have spontaneously evolved resistance to glyphosate in recent decades (Powles & Preston, 2006; Heap, 2009). In *Amaranthus palmeri*, for example, resistance is attributable to increased EPSPS expression resulting from multiple copies of the *epsps* gene, known as gene amplification; extra copies of the *epsps* gene were found on every chromosome (Gaines *et al.*, 2010). A similar gene amplification mechanism for overproduction of EPSPS was reported in *Lolium perenne* ssp. *multiflorum* (Salas *et al.*, 2012). Gaines *et al*. (2010) speculated that increased EPSPS expression in resistant biotypes could have a fitness penalty, but it seems possible that some resistant biotypes could have enhanced fitness, similar to the results reported here. Clearly, both scenarios deserve further investigation.

Other mechanisms for resistance to glyphosate have been attributed to traits that restrict translocation of the herbicide or mutations of the *epsps* gene that prevent inhibition by glyphosate, rather than overproduction of EPSPS (Xu *et al.*, 2002; Powles & Preston, 2006). However, it seems possible that several mechanisms for glyphosate resistance could evolve in the same species, together or separately, and perhaps in different regions (Powles *et al.*, 1997; Dinelli *et al.*, 2008). If so, selection favoring greater expression of a plant's endogenous *epsps* gene(s) could complement or substitute for other mechanisms. This could occur as a result of any genetic mechanism that leads to overproduction of EPSPS, perhaps causing downstream fitness benefits, as observed in the current study.

Previous studies of several glyphosate-resistant weeds offer preliminary support for this hypothesis. Increased levels of EPSPS mRNA have been reported in glyphosate-resistant biotypes of *Conyza* spp. (Dinelli *et al.*, 2006, 2008) and rigid ryegrass (*Lolium rigida*) (Baerson *et al.*, 2002). Shrestha *et al*. (2010) reported faster growth and greater competitive ability of glyphosate-resistant biotypes of *Conyza canadensis* when compared with susceptible biotypes in California in the absence of glyphosate treatments, although possible effects of genetic background were not accounted for in these studies (Vila-Aiub *et al.*, 2009). Other studies also report faster growth and earlier flowering in glyphosate-resistant weeds, but the genetic mechanisms and fitness consequences of earlier development require further research (Westhoven *et al.*, 2008 for *Chenopodium album*; Brabham *et al.*, 2011 for *Ambrosia trifida*). Our hypothesis that evolved resistance to glyphosate may enhance fitness when plants are not exposed to this herbicide should be tested using rigorously designed field experiments that compare the lifetime fitnesses of resistant and susceptible biotypes from the same genetic background, following protocols recommended by Vila-Aiub *et al*. (2009).

In conclusion, our results suggest that the overproduction of EPSPS can significantly increase plant growth and seed production in crop–weed hybrids of rice. To our knowledge, this is one of the first demonstrations of a strong fitness benefit associated with resistance to a herbicide *per se* (i.e. not dependent on exposure; also see Wang *et al.*, 2010). Our findings have broad implications for plant biology, crop breeding, weed management, biotech risk assessment, and the ongoing evolution of herbicide-resistant weeds. Further research is needed to examine which glyphosate-resistant crops and weeds may have acquired enhanced levels of EPSPS and, if so, the extent to which overproduction of EPSPS affects crop yields and weed fitness.

## Supporting Information

Table S1 Weedy rice (*Oryza sativa* f. *spontanea*) biotypes and cultivated rice (*Oryza sativa*) lines used for the production of crop–weed hybrids [Correction made after online publication 5 September 2013. Table S1 substituted for a version which explains the difference between EP3 and Minghui-86, and which illustrates that the transgenic EP3 produced more tillers than the control in a pilot experiment.]

Table S2 Methodology for measuring the fitness-related traits

Table S3 Effects of the *epsps* transgene on fitness-related traits in F_1_ and F_2_ crop–weed hybrids (WH1–WH4) from four biotypes of weedy rice (*Oryza sativa* f. *spontanea*) (W1–W4)
