# Supplementary material for: A novel 5-enolpyruvoylshikimate-3-phosphate (EPSP) synthase transgene for glyphosate resistance stimulates growth and fecundity in weedy rice (Oryza sativa) without herbicide
Source: New Phytol. 2013 Aug 1;202(2):679–88. doi: 10.1111/nph.12428 (PMC4286024; doi:10.1111/nph.12428)
Supplement: Table S3 — Effects of the epsps transgene on fitness-related traits in F1 and F2 crop–weed hybrids (WH1–WH4) from four biotypes of weedy rice (Oryza sativa f. spontanea) (W1–W4) [file nph0202-0679-SD1.doc]

**Supporting Information Tables S1–S3**

**Table S1** Weedy rice biotypes and cultivated rice lines used for the production of crop-weed hybrids, with information on their origin, morphological traits, and approximate days to flowering from seed germination. Data were obtained from a pilot experiment conducted in the Biosafety Assessment Centers in Fuzhou, Fujian Province of China, in 2009. For the two cultivated rice lines, mean plant height and number of tillers were calculated from averages taken from 36 plants in each of four replicate plots (N = 4). Numbers in parentheses following the means indicate standard errors. Means were compared between transgenic EP3 and non-transgenic Minghui-86 using independent t-tests; * P < 0.05. Note that the EP3 transgenic line produced significantly more tillers per plant than the non-transgenic control.

| Plant material |  | Country of origin | Plant height | No. of tillers | Days to flowering |
| --- | --- | --- | --- | --- | --- |
| Weedy Biotype W1 |  | Vietnam | ~140 | 12-17 | ~102 |
| Weedy Biotype W2 |  | South Korea | ~120 | 18-21 | ~95 |
| Weedy Biotype W3 |  | Nepal | ~150 | 12-14 | ~120 |
| Weedy Biotype W4 |  | China | ~150 | 11-13 | ~98 |
| CULTIVATED RICE LINES: |  |  |  |  |  |
| EP3 (Minghui-86 with *epsps* transgene) |  | China | 119.4 (1.5) | 19.0 (1.1)* | ~101 |
| Minghui-86 (nontransgenic) |  | China | 118.8 (1.0) | 14.3 (0.9) | ~100 |

**Table S2** Methodology for measuring the fitness-related traits

| Trait | Measurement |
| --- | --- |
| Plant height (cm) | Measured from the base of plants at the ground to the tips of the tallest panicles at maturity. |
| Number of tillers per plant | Total number of tillers from each plant measured at the tillering stage (~70 d after seed germination). |
| Number of panicles per plant | Total number of panicles from each plant measured at maturity (~120–140 d after seed germination). |
| Number of seeds per plant | Total number of well-developed seeds from each plant measured at maturity. |
| Seed set (%) | (Number of well-developed seeds per plant / total number of florets plant) ×100. |
| 1000-seed weight (g) | (Weight of total number of seeds per plant / total number of seeds per plant) ×1000. The seed weight was measured after seeds were dried at room temperature (~25^o^ C) for 60 d and then in an oven (~55^o^ C) for 5 d. |
| Seed germination (%) | (Number of germinated seeds / total number of seeds measured) ×100. Seeds were germinated for 4 d in an incubator at 37^o^ C, 90 d after harvest. Only seeds from the F_3_ generation were included. |

**Table S3** Effects of the *epsps* transgene on fitness-related traits in F_1_ and F_2_ crop-weed (*Oryza sativa* f. *spontanea* and *O. sativa*) hybrids (WH1–WH4) from four biotypes of weedy rice (W1–W4)

**A.**  WH1

| Trait | WH1 | | | |
| --- | --- | --- | --- | --- |
|  | Pure GE | Pure non-GE | Mixed GE | Mixed non-GE |
| **F_1_ hybrids (2009)** |  |  |  |  |
| Plant height (cm) | 147.2±2.7 | 140.2±2.2 | 144.1±3.8* | 138.8±4.3 |
| No. of tillers per plant | 22.7±1.1** | 19.3±0.3 | 22.6±0.7** | 12.7±0.7 |
| No. of panicles plant | 14.6±0.3** | 12.6±0.2 | 16.4±0.2** | 9.3±0.7 |
| No. of seeds per plant | 860.2±16.3** | 736.0±24.1 | 855.1±24.4** | 596.9±23.1 |
| Seed set | 55.4±3.3 | 44.8±4.2 | 49.3±3.8 | 45.2±1.9 |
| 1000-seed weight (g) | 24.1±0.6 | 24.3±0.3 | 23.5±0.7 | 22.9±0.3 |
|  |  |  |  |  |
| **F_2_ hybrids (2011)** |  |  |  |  |
| Plant height (cm) | 131.2±2.3 | 134.0±2.4 | 134.5±8.6 | 132.0±8.2 |
| No. of tillers per plant | 21.2±0.7** | 18.5±1.1 | 20.6±0.6* | 17.1±1.8 |
| No. of panicles per plant | 18.0±1.1** | 15.4±1.2 | 17.0±0.6* | 14.5±0.9 |
| No. of seeds per plant | 1198.0±50.2** | 770.5±45.7 | 966.6±32.7** | 523.1±36.9 |
| Seed set | 58.9±3.9 | 56.4±3.4 | 55.6±4.3 | 57.3±5.1 |
| 1000-seed weight (g) | 25.6±0.3 | 25.5±0.2 | 26.4±0.5 | 24.9±0.2 |

**B.** WH2

| Trait | WH2 | | | |
| --- | --- | --- | --- | --- |
|  | Pure GE | Pure non-GE | Mixed GE | Mixed non-GE |
| **F_1_ hybrids (2009)** |  |  |  |  |
| Plant height (cm) | 141.6±1.2 | 150.8±1.8** | 142.8±3.5 | 150.7±2.9 |
| No. of tillers per plant | 28.6±0.4** | 23.2±0.7 | 21.7±1.1* | 14.2±0.6 |
| No. of panicles per plant | 18.1±0.1* | 16.2±0.4 | 16.9±0.5* | 14.3±0.8 |
| No. of seeds per plant | 836.5±11.7*** | 642.6±17.0 | 857.8±12.7*** | 615.4±10.3 |
| Seed set | 60.1±7.6* | 36.2±3.4 | 52.0±2.9 | 45.4±3.2 |
| 1000-seed weight (g) | 21.9±0.1 | 21.3±0.5 | 22.6±0.6 | 22.1±0.4 |
|  |  |  |  |  |
| **F_2_ hybrids (2011)** |  |  |  |  |
| Plant height (cm) | 124.0±2.2 | 124.9±0.4 | 127.6±9.6 | 127.9±11.6 |
| No. of tillers per plant | 28.6±0.4** | 23.2±0.7 | 22.6±1.7** | 17.4±1.2 |
| No. of panicles per plant | 23.9±0.6** | 17.8±0.8 | 18.7±2.2** | 14.3±1.8 |
| No. of seeds per plant | 1166.4±27.5*** | 744.0±65.5 | 1049.7±22.9*** | 547.0±12.0 |
| Seed set | 66.0±2.5* | 54.8±3.0 | 63.8±2.4* | 54.5±2.0 |
| 1000-seed weight (g) | 23.7±0.4 | 23.6±0.2 | 23.1±0.4 | 23.5±0.4 |

**C.**  WH3

| Trait | WH3 | | | |
| --- | --- | --- | --- | --- |
|  | Pure GE | Pure non-GE | Mixed GE | Mixed non-GE |
| **F_1_ hybrid (2009)** |  |  |  |  |
| Plant height (cm) | 146.5±1.9 | 150.3±2.4 | 151.2±2.4 | 149.1±5.2 |
| No. of tillers per plant | 20.7±0.3** | 18.5±0.4 | 20.6±1.0** | 12.4±0.9 |
| No. of panicles per plant | 15.8±0.1* | 11.2±0.8 | 15.8±0.3* | 11.0±0.9 |
| No. of seeds per plant | 853.8±9.7*** | 641.5±11.0 | 847.3±16.7** | 564.0±32.3 |
| Seed set | 57.6±2.9* | 47.3±1.6 | 56.7±2.5* | 39.8±1.5 |
| 1000-seed weight (g) | 21.6±0.6 | 21.3±0.1 | 21.8±0.3 | 21.3±0.2 |
|  |  |  |  |  |
| **F_2_ hybrids (2011)** |  |  |  |  |
| Plant height (cm) | 144.1±3.6 | 139.8±8.0 | 146.5±5.2 | 146.5±3.5 |
| No. of tillers per plant | 18.6±1.1** | 14.1±0.5 | 16.6±2.0** | 11.1±0.7 |
| No. of panicles per plant | 15.1±0.5** | 11.5±0.4 | 13.6±1.8** | 9.0±1.2 |
| No. of seeds per plant | 1072.5±38.3** | 725.4±34.4 | 925.3±38.6*** | 412.1±29.2 |
| Seed set | 49.1±5.1 | 49.8±2.6 | 49.2±3.6 | 44.2±2.5 |
| 1000-seed weight (g) | 23.8±0.3 | 23.5±0.5 | 23.6±0.4 | 22.6±0.5 |

**D.** WH4

| Trait | WH4 | | | |
| --- | --- | --- | --- | --- |
|  | Pure GE | Pure non-GE | Mixed GE | Mixed non-GE |
| **F_1_ hybrid (2009)** |  |  |  |  |
| Plant height (cm) | 144.6±6.8 | 147.0±2.6 | 152.8±3.1 | 139.9±5.4 |
| No. of tillers per plant | 18.1±0.9** | 12.6±0.5 | 16.4±1.0* | 11.3±0.7 |
| No. of panicles per plant | 11.0±0.3*** | 8.5±0.08 | 10.9±0.2* | 8.1±0.4 |
| No. of seeds per plant | 766.6±13.8** | 646.9±18.4 | 772.8±15.7*** | 538.0±6.8 |
| Seed set | 50.3±4.8 | 45.8±1.9 | 53.4±3.4 | 47.5±3.7 |
| 1000-seed weight (g) | 22.9±0.4 | 21.3±0.9 | 20.9±0.2 | 20.0±1.0 |
|  |  |  |  |  |
| **F_2_ hybrids (2011)** |  |  |  |  |
| Plant height (cm) | 128.4±2.3 | 127.7±1.8 | 129.0±11.0 | 130.9±11.2 |
| No. of tillers per plant | 16.1±1.2** | 12.4±0.4 | 15.2±2.1** | 11.0±0.6 |
| No. of panicles per plant | 13.1±1.3** | 10.6±1.3 | 12.0±1.1*** | 8.5±0.8 |
| No. of seeds per plant | 1030.3±28.0** | 663.1±27.0 | 966.6±32.6*** | 523.1±37.0 |
| Seed set | 59.6±1.6 | 57.0±1.9 | 55.0±1.7 | 52.0±0.8 |
| 1000-seed weight (g) | 24.8±0.2 | 24.5±0.4 | 25.5±0.2 | 24.2±0.3 |

Plants were grown in the field experiments under pure and mixed cultivation (see text). Means ± SE are shown; *n* = 4 plots for F_1_ (2009) and 6 plots for F_2_ (2011). Comparisons were made between GE and non-GE crop-weed plants in pure cultivation using independent *t*-tests and in mixed cultivation using paired *t*-tests, with Bonferonni corrections. *, *P* < 0.05; **, *P* < 0.01; ***, *P* < 0.001.
